# Hyperstable EGF-like bleogen derived from cactus accelerates corneal healing in rats

**DOI:** 10.3389/fphar.2022.942168

**Published:** 2022-08-16

**Authors:** Shining Loo, Antony Kam, James P. Tam

**Affiliations:** School of Biological Sciences, Nanyang Technological University, Singapore, Singapore

**Keywords:** bleogen, cactus, corneal wound healing, epidermal growth factor, myofibroblast, opacity, vision

## Abstract

Corneal scarring reduces corneal transparency, compromises vision, and is a major cause of vision loss worldwide. Epidermal growth factor (EGF), which is the prototypic member of the EGF receptor (EGFR) agonists, is present in tears to provide repair and regeneration. Recently, we discovered bleogen pB1 in the cactus plant *Pereskia bleo* and showed that it is a non-canonical and hyperstable EGFR agonist with EGF-like wound healing properties for diabetic rats. Here, we apply bleogen pB1 to accelerate corneal wound healing in rats. To assess the corneal healing effects of bleogen pB1, we induced an acute alkali burn to the right eye of male Wistar rats. After five consecutive ophthalmic applications, fluorescein staining and opacity scores of the bleogen pB1-treated, and the positive control EGF-treated groups improved significantly compared to the saline control group. Immunohistochemical analyses revealed that infiltrated CD68^+^ macrophages and the expression of the myofibroblast marker alpha smooth muscle actin (α-SMA) were significantly decreased in the bleogen pB1- and the EGF-treated groups. By employing a differential gene expression analysis of bleogen pB1- and EGF-treated keratinocytes through RNA-seq, we demonstrated that bleogen pB1 or EGF treatments can affect the expression of genes associated with inflammatory responses and extracellular matrix remodeling. Taken together, our results indicate that the plant-derived EGFR agonist bleogen pB1 can produce similar effects to those of EGF in accelerating corneal wound healing as well as in reducing persistent inflammation and myofibroblast accumulation in the cornea.

## Introduction

The epidermal growth factor (EGF) is a 53-amino acid miniprotein that was discovered in 1960 (Cohen and Levi-Montalcini, 1956; Cohen, 1960, 1962). EGF recognizes and binds to the EGF receptor (EGFR/ErbB1/HER) on cell surfaces (Cohen, 1960; Lu et al., 2001; Singh et al., 2016). This binding is known to stimulate a ligand-induced receptor dimerization and activation of the intrinsic protein-tyrosine kinase activity, which, in turn, initiates a signal transduction cascade (Carpenter et al., 1978; Cohen et al., 1980; Isobe et al., 1995). The resulting biochemical changes within the cell ultimately lead to cell proliferation, survival, and differentiation (Singh et al., 2016). To date, seven related mammalian EGFR agonists (ranging from 46 to 132 residues) have been identified, including EGF, the transforming growth factor-alpha (TGF-α), the heparin-binding epidermal growth factor (hb-EGF), betacellulin, amphiregulin, epiregulin, and epigen (Cohen, 1962; Todaro and De Larco, 1978; Shoyab et al., 1988; Shoyab et al., 1989; Higashiyama et al., 1991; Shing et al., 1993; Isobe et al., 1995; Strachan et al., 2001; Singh et al., 2016). All EGF-related family members contain a typical EGF motif with a two-subdomain structure consisting of an N-terminal A-/B-loop formed by the first two disulfide bonds, and a C-terminal C-loop formed by the third disulfide bond (Lu et al., 2001).

**FIGURE 1:**
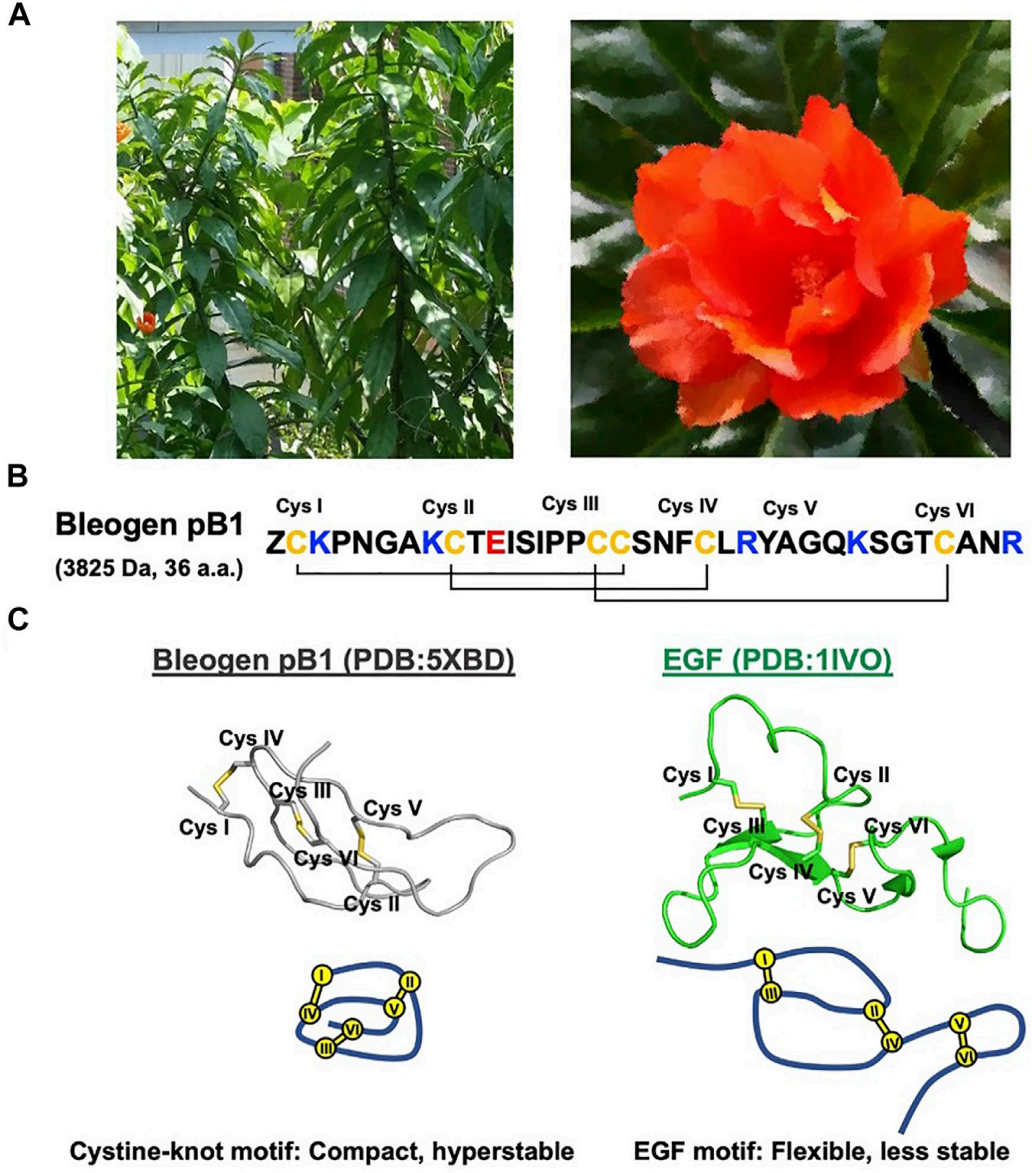
Bleogen pB1 is a cystine knot peptide deriving from *Pereskia bleo* and characterized by epidermal growth factor (EGF)-like activities. **(A)** Leaves and flowers of *Pereskia bleo*. **(B)** Primary sequence of bleogen pB1, in which “Z” represents pyroglutamate. **(C)** Disulfide connectivity and tertiary structural differences between bleogen pB1 and EGF.

Recently, our laboratory discovered and identified the first plant-derived EGFR agonist known as bleogen pB1 (Loo et al., 2021a). Bleogen pB1 is a 36-residue miniprotein and the smallest known EGFR agonist. This positively charged bleogen pB1 was originally identified as a heparin-binding, antifungal, cysteine-rich peptide from the Southeast Asian cactus *Pereskia bleo* (Kunth) DC of the Cactaceae family (Loo et al., 2017) (Figure 1). The inspiration for its discovery as an EGFR agonist originated from the traditional use of *Pereskia bleo* in wounds and related ailments, including ulcers, gastritis, haemorrhoids, cold sore, and atopic dermatitis (Abdul-Wahab et al., 2012; Sharif et al., 2013; de Castro Campos Pinto and Scio, 2014; Zareisedehizadeh et al., 2014; Zareisedehizadeh et al., 2022). This is because EGF is clinically approved in several countries for treating chronic wounds such as diabetic foot ulcers (Brown et al., 1989; Harding et al., 2002; Gurtner et al., 2008; Bui et al., 2019). Hence, bleogen pB1 could be a bioactive principle in *Pereskia bleo* responsible for its traditional use in wound healing.

Bleogen pB1 contains a single domain stabilized by three disulfide bonds and two antiparallel β strands that form a cystine-knot, a disulfide arrangement that distinguishes it from all other known EGFR agonists. As such, bleogen pB1 is a non-canonical and “first-in-class” EGFR agonist. Although both the sequence and the structure of bleogen pB1 are different from those of EGF (Figure 1C), bleogen pB1 binds to and activates EGFR, and also displays functions that are similar to those of EGF *in vitro* (Loo et al., 2021a). Moreover, like EGF, bleogen pB1 promotes skin wound healing *in vivo* (Loo et al., 2021a). More importantly, bleogen pB1 is more than 100 times more resistant to proteolytic degradation than EGF. In fact, the proteolytic resistance of bleogen pB1 makes it less susceptible to inactivation by the proteases found in wounds, and this is an advantage over EGF and other EGFR agonists.

The cornea is a dedicated transparent avascular tissue in the eye that acts as a protective structural barrier; it also contributes to the refractive and focusing power of the eye (Bukowiecki et al., 2017; Peterson and Ceresa, 2021). However, the cornea is often exposed to the outer environment, thereby making it prone to insults such as physical and chemical injuries as well as infections. In fact, even small abrasions to the cornea, such as those caused from fingernails and contact lenses, can lead to glared or blurred vision and ultimately result in permanent vision loss that requires a corneal replacement (Bukowiecki et al., 2017).

Tears are of fundamental importance in maintaining overall eye health as they are known to contain a complex chemical cocktail of vitamins, immunoglobulins, proteases, antimicrobial peptides, growth factors, and electrolytes (You et al., 2010; Pflugfelder and Stern, 2020). Tears keep eyes moist and lubricated, thereby enabling them to remove irritants and foreign microorganisms. More importantly, tears provide oxygen, nutrients, and growth factors to the underlying avascular tissues in order for the latter to maintain tissue homeostasis (Pflugfelder and Stern, 2020). EGF is one of a half a dozen growth factors identified in tears, that is, particularly important for corneal wound healing (Van Setten et al., 1989; Pflugfelder and Stern, 2020). Several studies focusing on tear fluids isolated from healthy human volunteers indicated that the EGF concentration is approximately 2 ng/ml under normal conditions (Ohashi et al., 1989; van Setten et al., 1990; Van Setten et al., 1994; Nava et al., 1997; Peterson et al., 2014). Interestingly, the EGF concentration in tears rapidly increases after epithelial wounding in rabbits (Sheardown and Cheng, 1996). Previous studies have also demonstrated the importance of EGFR activation for the EGF-mediated corneal epithelial wound healing in various animal models (Singh and Foster, 1987; Zieske et al., 2000; Peterson et al., 2014), as well as the potential clinical use of EGF for the management of corneal ulcers in humans (Daniele et al., 1979; Pastor and Calonge, 1992).

In this study, we adopt the traditional usage of *Pereskia bleo* in ulcers, and apply it to corneal wound which is a leading cause of vision loss worldwide. Here, we show that the plant-derived bleogen pB1 accelerates corneal wound healing and reduces prolonged inflammation and myofibroblast accumulation after an acute alkaline burn *in vivo*.

## Results

### Solid-phase peptide synthesis and oxidative folding of the plant-derived EGFR agonist, bleogen pB1

**FIGURE 2:**
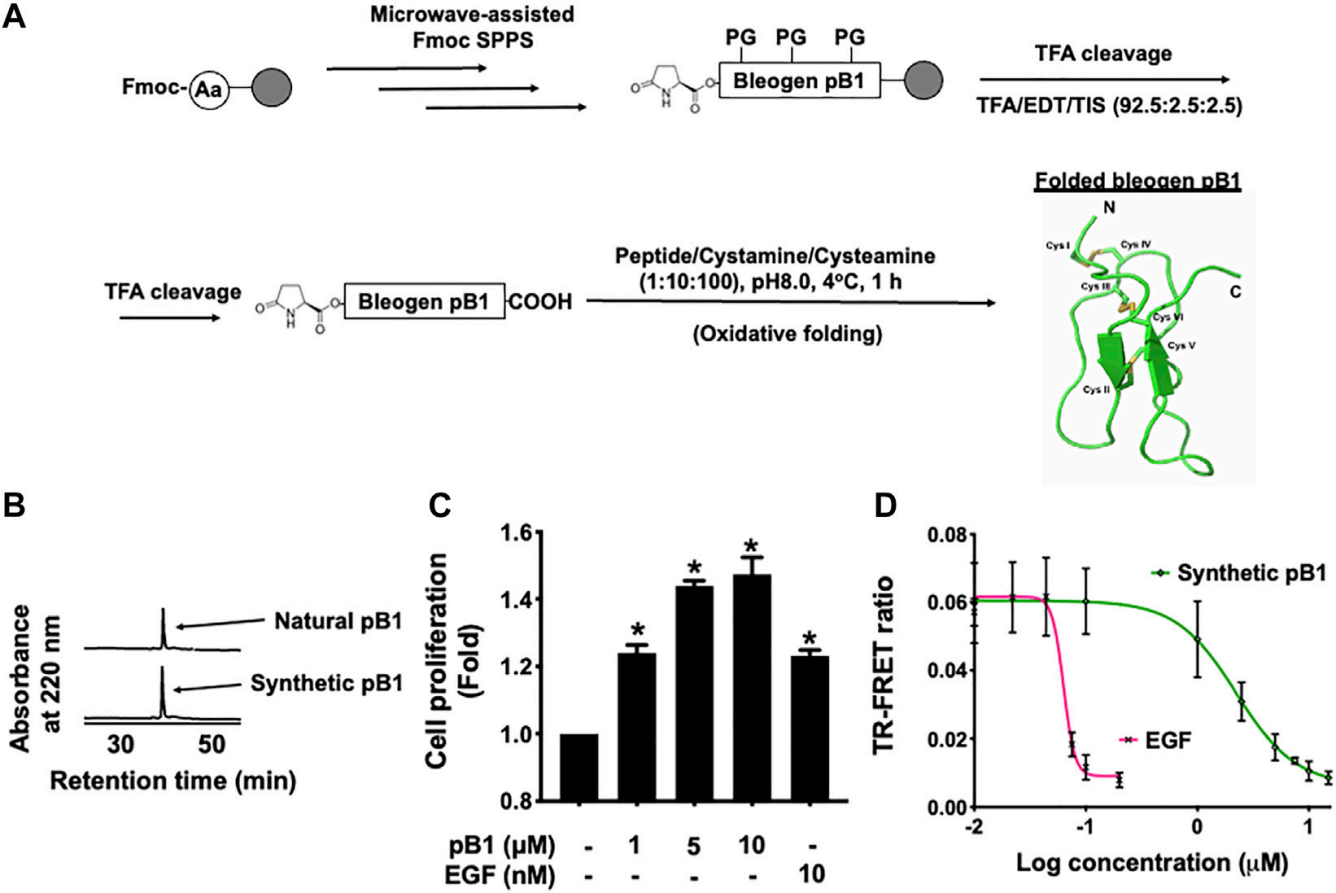
**(A)** Schematic overview of the chemical synthesis and oxidative folding of bleogen pB1 with N-terminal pyroglutamate. Synthetic bleogen pB1 with N-terminal pyroglutamate was synthesized by employing microwave-assisted solid-phase Fmoc chemistry on an HMPB-ChemMatrix resin (PG: Protecting Group). After trifluoroacetic acid (TFA) cleavage, the assembled linear precursor was released from the resin support and was subjected to oxidative folding in 0.1 M ammonium bicarbonate (at pH 8.0) and 10% dimethyl sulfoxide (DMSO) with a mixture of the redox reagents cysteamine/cystamine (at a 10:1 M ratio) for 1 h. **(B)** Elution of natural and synthetic bleogen pB1 by using reversed-phase high-performance liquid chromatography (RP-HPLC) **(C)** Effects of synthetic bleogen pB1 and epidermal growth factor (EGF) on HaCaT cells for 72 h, as assessed by using a crystal violet assay. All results are expressed as the mean ± standard deviation (SD); *: *p* < 0.05 as compared with the control. **(D)** Time-resolved fluorescence energy transfer (TR-FRET)-based competitive displacement of biotin-EGF from the EGF receptor (EGFR) after using synthetic bleogen pB1 or EGF (positive control). All results are expressed as the mean ± SD of three independent experiments.

In all experiments, we used synthetic bleogen pB1 produced by chemical synthesis, as previously described (Loo et al., 2021a). The linear precursor of this miniprotein with an N-terminal pyroglutamate was prepared by employing an Fmoc-based stepwise and microwave-assisted solid-phase synthesis (Figure 2A). The linear precursor was then cleaved from the resin support by trifluoroacetic acid (TFA) and was oxidatively folded using a combination of redox reagents including cysteamine and cystamine (at a 10:1 M ratio) in 0.1 M ammonium bicarbonate (pH 8) for 1 h, to yield bleogen pB1. The identity, integrity, and function of the synthetic bleogen pB1 were confirmed via mass spectrometry analysis, reversed-phase high-performance liquid chromatography (RP-HPLC) co-elution with the native bleogen pB1, cell proliferation assays, as well as time-resolved fluorescence energy transfer (TR-FRET)-based EGFR competitive displacement studies (Figures 2B–D; Supplementary Figure S1).

### Bleogen pB1 accelerates corneal wound healing and reduces cornea opacity following acute alkali-induced burns in rats

**FIGURE 3:**
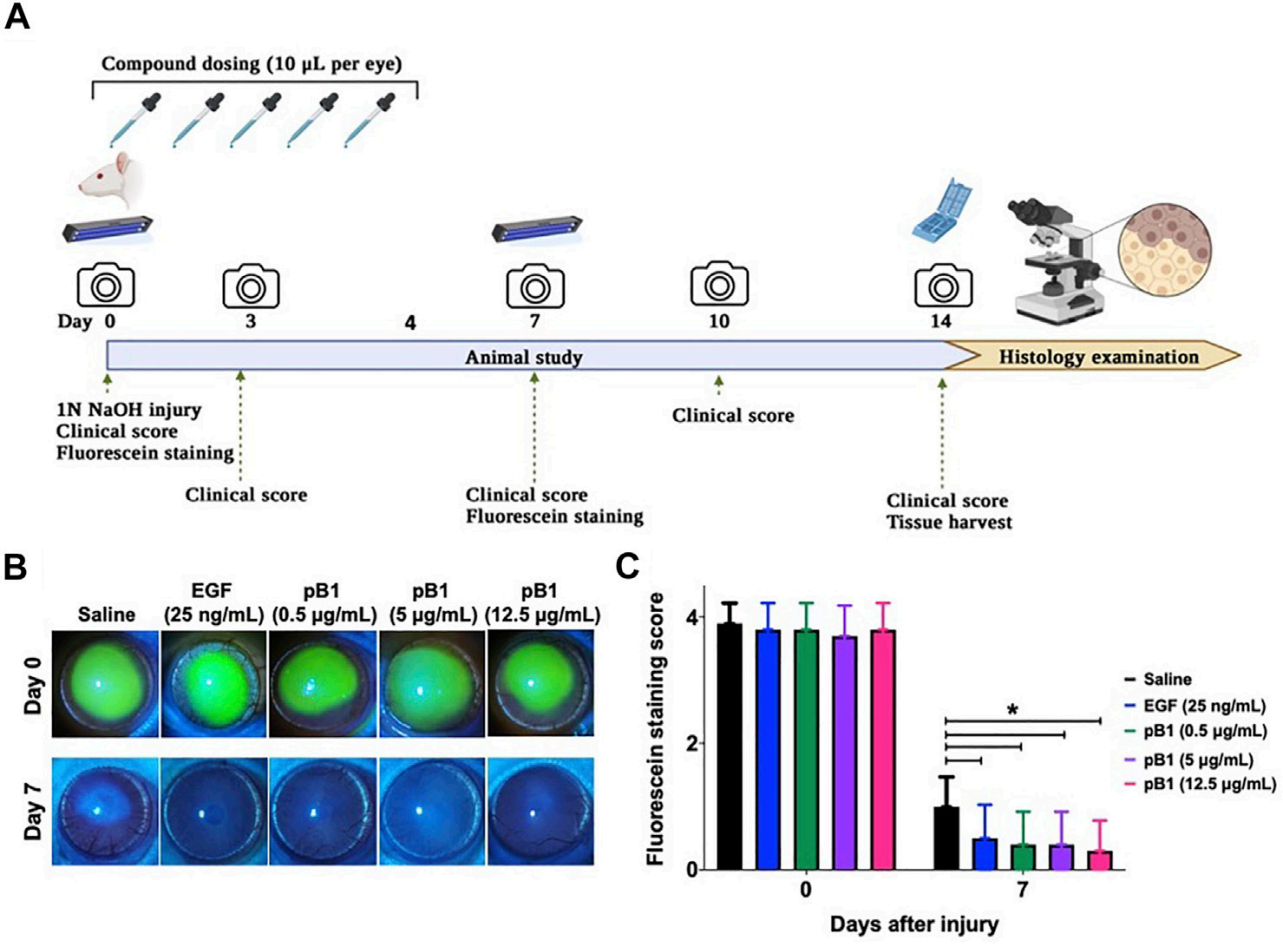
Bleogen pB1 accelerates corneal wound healing in male Wistar rats after a burn caused by NaOH. **(A)** Treatment regimen of the acute corneal alkali injury model. In total, 50 rats were subjected to acute alkali burn injury to the right eye by placing a piece of 3.2-mm-diameter filter paper soaked in 1 N NaOH for 30 s on the eye. After the injury, the rats were separated into five groups: (1) saline control (10 rats), (2) EGF (25 ng/ml; 10 rats), (3) pB1 (0.5 μg/ml; 10 rats), (4) pB1 (5 μg/ml; 10 rats), and (5) pB1 (12.5 μg/ml; 10 rats). Ophthalmic application of the test compounds was performed for five consecutive days after the induction of the injury. Fluorescein staining was performed on days 0 and 7 after the injury in order to assess the corneal epithelial damage. On days 0, 3, 5, 7, 10, and 14 after the injury, both the corneal opacity and the neovascularization were examined. **(B)** Representative photographs of the wound area on days 0 and 7 after the injury as defined through fluorescein staining. **(C)** Bar charts showing the fluorescein staining score of all corneal injury groups. All results are expressed as the mean ± standard deviation (SD); *p < 0.05 for all treated groups, as compared with the saline control group. Notes: EGF: epidermal growth factor.

**FIGURE 4:**
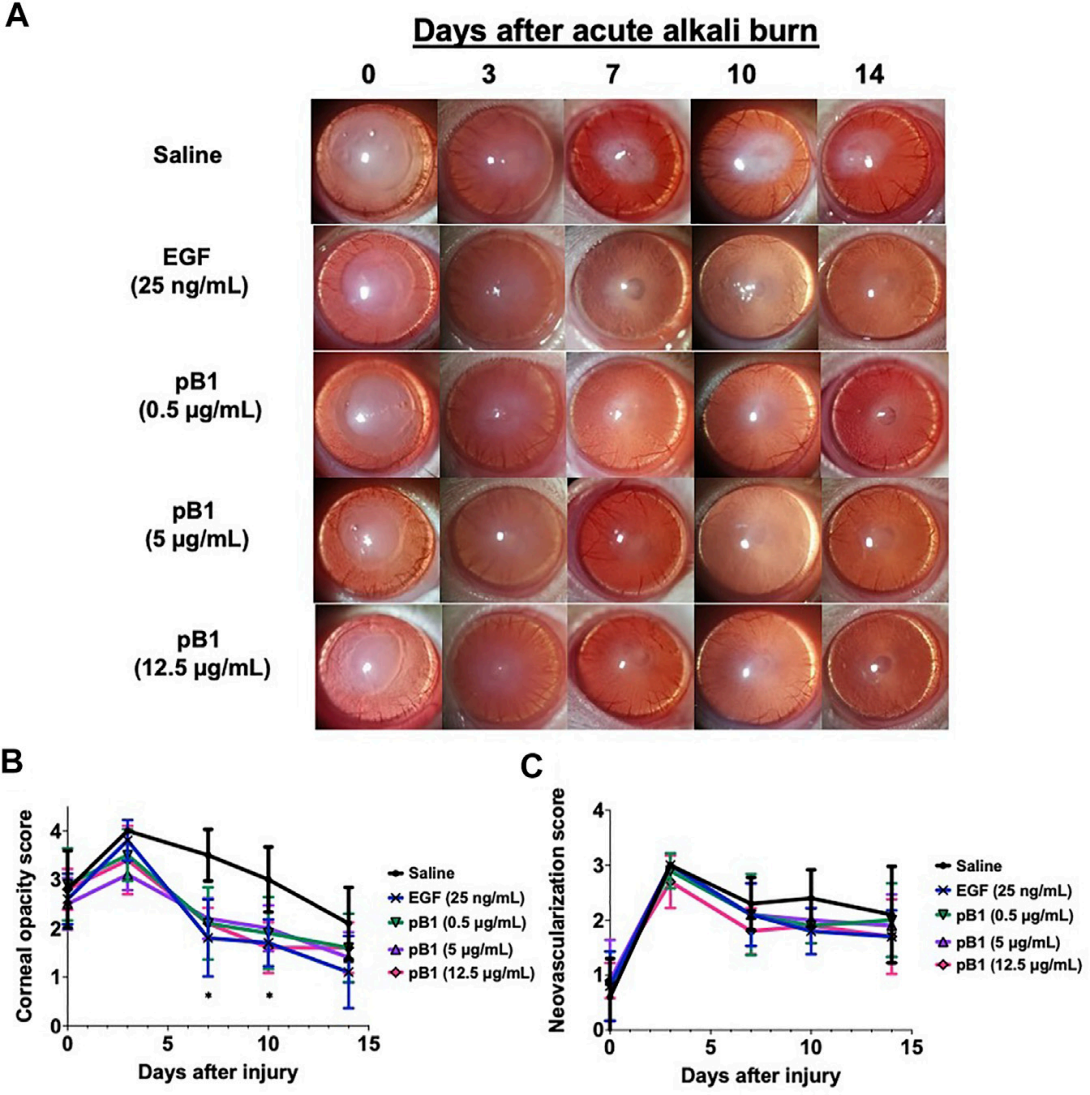
Bleogen pB1 reduces corneal opacity in male Wistar rats after a burn caused by 1-N NaOH. **(A)** Representative photographs of the wound area on days 0, 3, 7, 10, and 14 after the induction of the injury in all corneal injury groups. Five corneal injury groups: (1) saline control (10 rats), (2) EGF (25 ng/ml; 10 rats), (3) pB1 (0.5 μg/ml; 10 rats), (4) pB1 (5 μg/ml; 10 rats), and (5) pB1 (12.5 μg/ml; 10 rats). **(B)** Bar charts showing the corneal opacity score in different groups. **(C)** Bar charts demonstrating the corneal neovascularization score of all corneal injury groups. All results are expressed as the mean ± standard deviation (SD); *: *p* < 0.05 for all treated groups, as compared with the saline control group. Notes: EGF: epidermal growth factor.

To show that bleogen pB1 could be used for corneal wound healing, we used an *in vivo* corneal alkali burn model. An acute alkaline burn was applied onto the right eye of male Wistar rats by placing a 3.2-mm-diameter filter paper soaked in 1 N NaOH on the eye for 30 s. Subsequently, the fluorescein staining, the opacity, and the neovascularization scores were assessed to monitor the process of corneal wound healing (Figure 3A). Consistent with previous reports, our results showed that the NaOH-induced burn caused a corneal epithelial damage, as shown by the positive fluorescein stain on day 0 (Figures 3B,C). The opacity score was noted to increase from day 0 and reach a peak on day 3 (Figures 4A,B). Similar trends were also observed in the case of the neovascularization score with a peak on day 3 (Figures 4A–C).

To determine if bleogen pB1 can accelerate corneal wound healing, bleogen pB1 (0.5, 5 or 12.5 μg/ml which corresponds to ∼ 0.13, 1.3, and 3.3 μM, respectively) or the positive control (EGF, 25 ng/ml) were ophthalmic-administered for five consecutive days after the alkali-induced burn injury (Figure 3A). On day 7, fluorescein staining was performed to assess corneal epithelial damage. The results indicated that bleogen pB1 and EGF significantly reduced the fluorescein staining score, as compared to the saline control group (Figures 3B,C). The opacity scores were also noted to significantly decrease after treatment with either bleogen pB1 or EGF on days 7 and 10 (Figures 4A,B). On the other hand, there are no significant changes to neovascularization score over the course of 14 days post injury when we compared the bleogen pB1- or EGF-treated with saline control group (Figures 4A–C).

**FIGURE 5:**
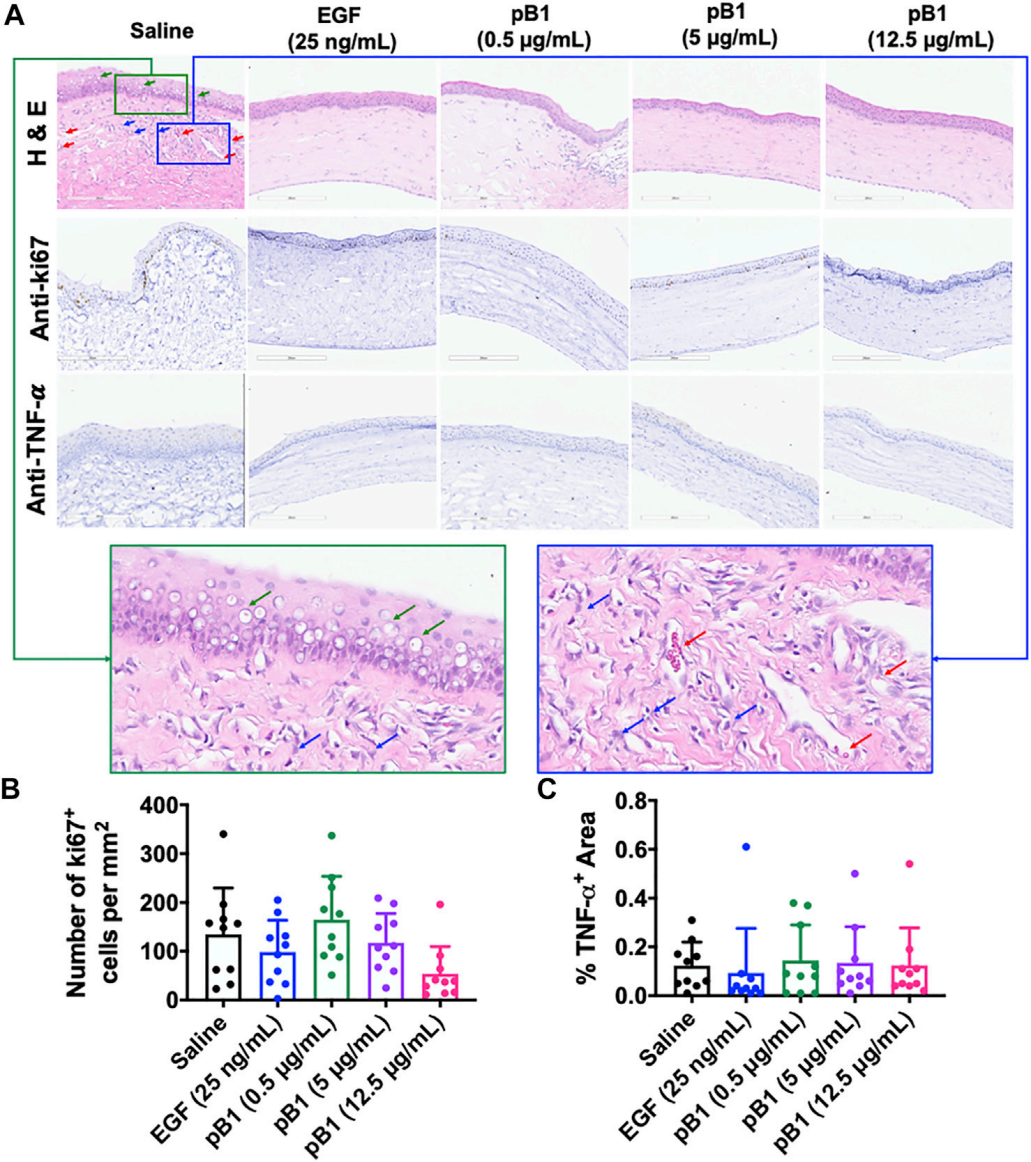
**(A)** Cross-sections of corneas harvested 14 days after the injury were stained with hematoxylin and eosin (in order to visualize the corneal tissue structure and the infiltration of inflammatory cells), and were immunostained for the proliferative marker Ki67 and the inflammatory marker tumor necrosis factor alpha (TNF-α). Green boxed area represents the magnified area with high level of corneal vacuole formation and inflammatory cell infiltration. Blue boxed area represents the magnified area with high level of inflammatory cell infiltration and blood vessel formation. Red arrow: blood vessels. Blue arrows: neutrophils. Green arrows: corneal vacuoles **(B,C)** Bar charts demonstrating **(B)** the number of Ki67-positive proliferating cells and **(C)** the TNF-α-positive areas of all corneal injury groups. Five corneal injury groups: (1) saline control (10 rats), (2) EGF (25 ng/ml; 10 rats), (3) pB1 (0.5 μg/ml; 10 rats), (4) pB1 (5 μg/ml; 10 rats), and (5) pB1 (12.5 μg/ml; 10 rats). All results are expressed as the mean ± standard deviation (SD); *: *p* < 0.05 for all treated groups, as compared with the saline control group. Notes: EGF: epidermal growth factor.

After day 14 of the alkali-induced burn injury, we harvested the rat corneas for histological analysis by hematoxylin and eosin (H&E) and immunohistochemical staining. As expected, the H&E staining revealed that epithelial closure was achieved in all examined groups (Figure 5A). The stained images of the saline control group however, displayed more corneal vacuoles, blood vessels, and neutrophils than the EGF- or the bleogen pB1-treated groups (Figure 5A). To determine the stages of corneal wound healing, immunohistochemical staining was performed using tumor necrosis factor-alpha (TNF-α) and Ki67 as markers for inflammation and proliferation, respectively. The results showed that there were no significant differences in the expressions of TNF-α and Ki67 for the saline-, bleogen pB1-, and EGF-treated groups, suggesting that the process of corneal wound healing has passed the inflammatory and proliferative phase after day 14 (Figures 5A–C).

### Bleogen pB1 reduces prolonged inflammatory cell infiltration and myofibroblast accumulation in the rat cornea

**FIGURE 6:**
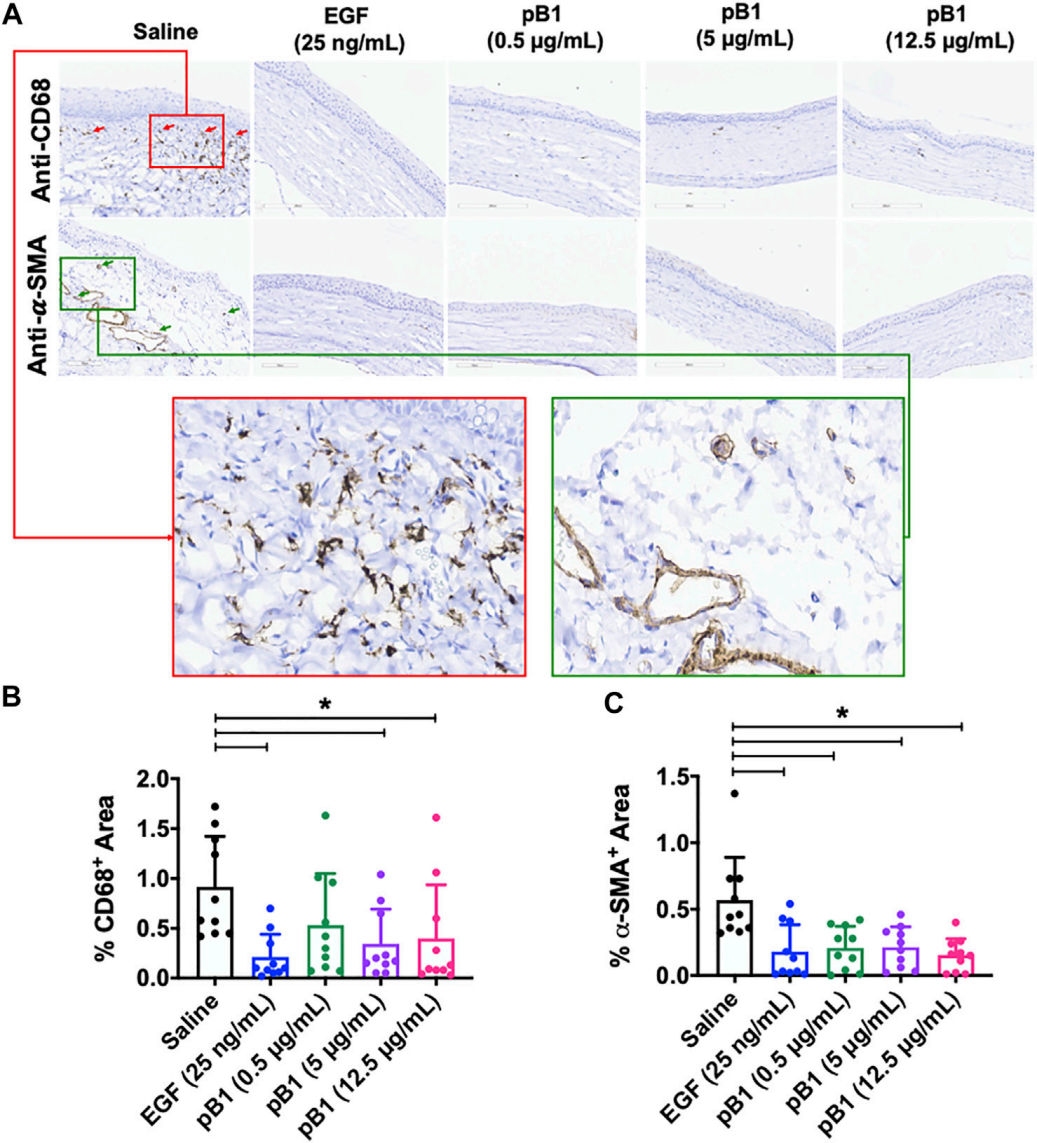
**(A)** Cross sections of corneas harvested at 14 days after injury were immunostained with antibodies for CD68 and alpha smooth muscle actin (α-SMA) in order to identify macrophages and myofibroblasts, respectively. Red boxed area represents the magnified area with high level of positive CD68 staining. Green boxed area represents the magnified area with high level of positive α-SMA staining **(B,C)** Bar charts demonstrating **(B)** the number of CD68-positive and **(C)** α-SMA-positive areas of all corneal injury groups. Five corneal injury groups: (1) saline control (10 rats), (2) EGF (25 ng/ml; 10 rats), (3) pB1 (0.5 μg/ml; 10 rats), (4) pB1 (5 μg/ml; 10 rats), and (5) pB1 (12.5 μg/ml; 10 rats). All results were expressed as the mean ± standard deviation (SD); *: *p* < 0.05 for all treated groups, as compared with the saline control group. Notes: EGF, epidermal growth factor.

Previous reports have shown that corneal opacity occurs when there is an excessive influx of inflammatory cells to the wound area, particularly immediately after the induction of an injury (Bukowiecki et al., 2017). However, prolonged inflammatory cell accumulation in the cornea can lead to abnormal corneal wound healing, scarring, and vision loss (Bukowiecki et al., 2017). To determine if bleogen pB1 can reduce persistent inflammatory cell infiltration, we analyzed our H&E-stained tissue sections. Our results showed that there were more neutrophils in the cornea of the saline control group than in the bleogen pB1- or EGF-treated groups (Figure 5A). We also performed immunohistochemical staining for CD63, a marker for macrophages. Our results showed that rat corneas treated with either bleogen pB1 or EGF hosted significantly lower number of CD63^+^ macrophages than the corneas of the saline control group (Figures 6A,B).

Another known factor for prolonged corneal opacity is the persistent accumulation of myofibroblasts in the cornea (Jester et al., 1999; Qazi et al., 2010). Previous reports have demonstrated that myofibroblasts can be differentiated from a variety of cell types, including keratinocytes, fibroblasts, endothelial cells, and stem cells, a process that involves the transforming growth factor-beta 1 (TGF-β1)/Smad signaling pathway. To determine whether bleogen pB1 reduces the prolonged accumulation of myofibroblasts in the rat cornea, we performed immunohistochemical staining for alpha smooth muscle actin (α-SMA), a marker for myofibroblasts. The results showed that the corneas treated with bleogen pB1 or EGF have significantly fewer α-SMA-positive areas than the corneas of the saline control group (Figures 6A–C).

### Differentially expressed gene analysis in keratinocytes after treatment with bleogen pB1 or EGF

**FIGURE 7:**
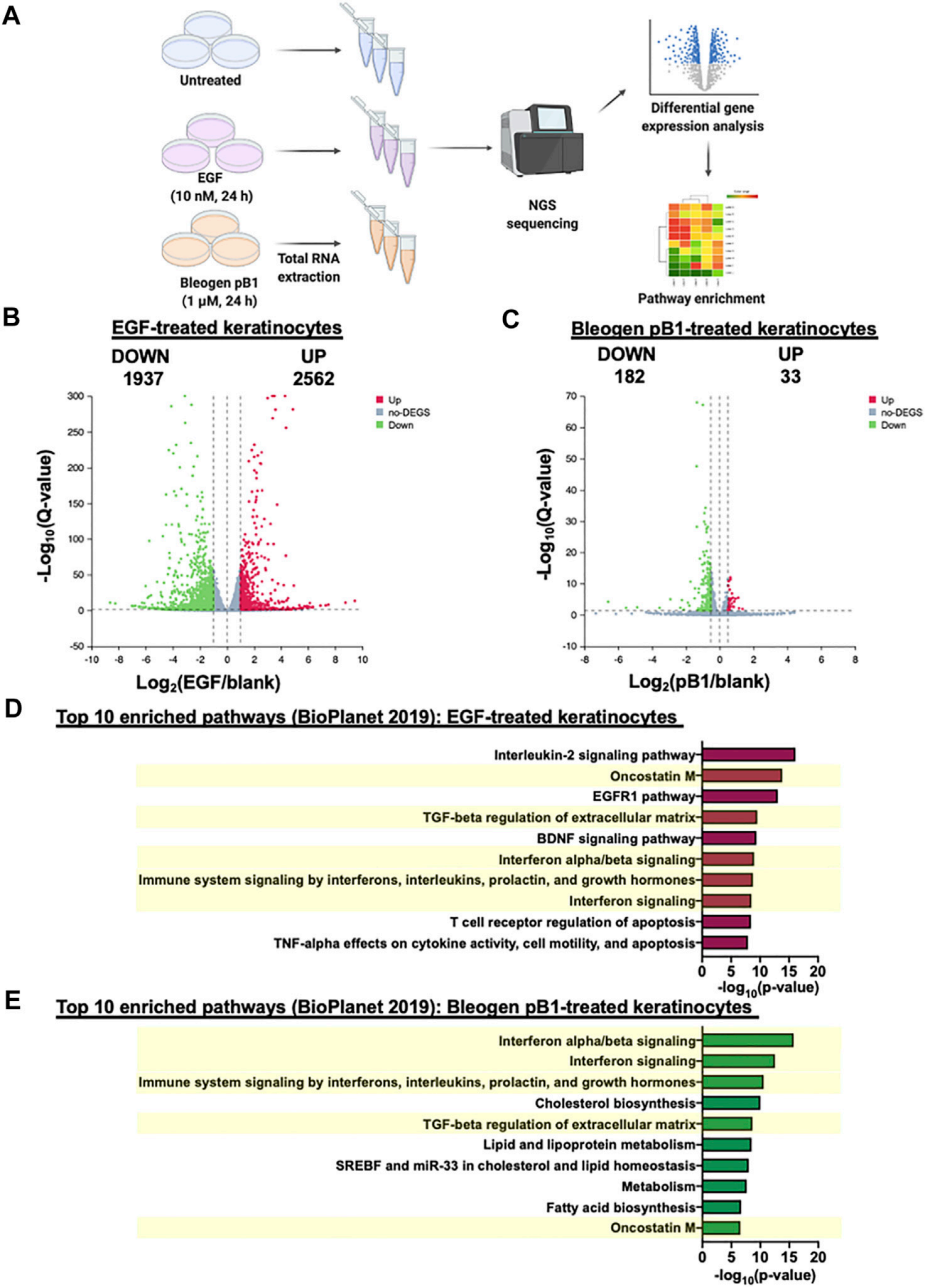
**(A)** Differential gene expression analysis based on the transcriptome profiles of HaCaT keratinocytes after treatment (*n* = 3) with bleogen pB1 (1 μM; 24 h) or epidermal growth factor (EGF) (10 nM; 24 h) **(B)** Volcano plot displaying the differentially expressed genes (DEGs) in HaCaT cells, as induced by an EGF treatment (10 nM) for 24 h **(C)** Volcano plot displaying the DEGs in HaCaT cells, as induced by a treatment with bleogen pB1 (1 µM) for 24 h **(D)** Pathway enrichment analysis of the differentially expressed HaCaT cell genes induced by an EGF treatment (10 nM; 24 h), as performed by using the BioPlanet 2019 database and the enrichR bioinformatics package **(E)** Pathway enrichment analysis of the differentially expressed HaCaT cell genes induced by a bleogen pB1 treatment (1 μM; 24 h), as performed by using the BioPlanet 2019 database and the enrichR bioinformatics package. The yellow box highlights the common pathways among the top 10 enriched pathways that were found to be shared between the EGF and the bleogen pB1 treatments.

To gain further insights into the wound healing effects of bleogen pB1, we performed transcriptome profiling using RNA-seq on bleogen pB1-treated, EGF-treated, and untreated HaCaT keratinocytes (Figure 7A). A total of 210,503 genes were detected. The principal component analysis revealed that the transcriptomes generated from the bleogen pB1-treated, the EGF-treated, and the untreated cells were well clustered and distinguishable from one another (Supplementary Figure S2). Differentially expressed genes (DEGs) were identified based on a DESeq2 analysis with the following significance thresholds: Q-value ≤ 0.05 and |log2FC|  ≥ 0.5, as compared to the untreated group. For bleogen pB1, a total of 215 DEGs were identified, of which 33 genes were upregulated and 182 genes were downregulated (Figure 7B, Supplementary Table S1). For EGF, a total of 4,499 DEGs were identified, of which 2,562 genes were upregulated and 1,937 genes were downregulated (Figure 7C, Supplementary Table S2).

To determine the shared pathways of bleogen pB1 and EGF, we performed a pathway enrichment analysis on the DEGs using the BioPlanet 2019 database along with the enrichR bioinformatics package (Chen et al., 2013; Huang et al., 2019). Figure 7D shows the top 10 enriched pathways for the EGF-treated keratinocytes, while Figure 7E shows the top ten enriched pathways for the bleogen pB1-treated keratinocytes. Of these enriched pathways, five are shared between the EGF- and the bleogen pB1-treated keratinocytes. They are namely the “TGF-β regulation of extracellular matrix,” the “interferon-alpha/beta signaling,” the “interferon signaling,” the “immune system signaling by interferons, interleukins, prolactin, and growth hormones,” and the “oncostatin M” pathways. The RNA-seq results suggest that in addition to their proliferative effects, both bleogen pB1 and EGF modulate the inflammatory and extracellular matrix remodeling/maturation phases during wound healing.

## Discussion

In this present study, we show that bleogen pB1 derived from *Pereskia bleo* can accelerate scar-free corneal wound healing in rats following an acute alkali burn injury. Bleogen pB1 was observed to exert comparable effects as EGF in the case of this ophthalmic application.

Corneal injury, often a result of physical, chemical, or infection-related traumas, has been identified as a major cause of permanent vision loss and blindness worldwide (Whitcher et al., 2001). Proper wound healing is essential for the maintenance of the structural integrity and transparency of the cornea and for the restoration of the visual function (Bukowiecki et al., 2017). Like wound healing of the skin, corneal wound healing is characterized by three highly integrated and overlapping phases: an inflammatory, a proliferative, and a maturation phase (Bukowiecki et al., 2017). Immediately after the induction of a corneal epithelial and stromal injury, a complex sequence of processes contributes to wound repair and the regeneration of the normal corneal structure, transparency, and function (Bukowiecki et al., 2017). The inflammatory phase involves the sequential infiltration of inflammatory cells aiming to remove cellular debris, damaged matrix, and microbes (Bukowiecki et al., 2017). This is followed by the proliferative phase that rebuilds tissues through cell migration, proliferation, and differentiation. Finally, the maturation phase increases the tensile strength of the wound through the undertaking of extracellular matrix remodeling (Bukowiecki et al., 2017). Depending on the type and extent of the injury, a prolonged influx of inflammatory cells, an imbalance in the myofibroblast turnover, and/or a disorganized extracellular matrix organization may occur, thereby resulting into corneal scarring and opacity (Bukowiecki et al., 2017).

Previously, we showed that both EGF and bleogen pB1 are mitogens that can accelerate incisor eruption in newborn mice and skin wound healing in streptozotocin-induced diabetic rats (Loo et al., 2021a). Similar to EGF, bleogen pB1 activates the EGFR/MEK/ERK signaling pathway in keratinocytes to trigger cell proliferation in healing skin wounds. Because EGF, which is present in tear film, is known to be safe and effective in reducing the healing time of corneal ulcers, we used an acute alkali burn rat model to compare the corneal healing effects of bleogen pB1 and EGF (Daniele et al., 1979; Singh and Foster, 1987; Pastor and Calonge, 1992; Van Setten et al., 1994). Like EGF, ophthalmic bleogen pB1-treatments accelerate corneal wound healing after an acute alkali-induced burn which is characterized by an improved corneal epithelial closure and corneal opacity scores. Also like EGF, bleogen pB accelerates corneal wound healing without increasing neovascularization score. This is particularly important because neovascularization can affect the corneal transparency. Additionally, both the corneas of the bleogen pB1- and EGF-treated groups exhibited low inflammatory cell infiltration and myofibroblast accumulation based on our H&E and immunohistochemical staining that could account for the marked improvement in corneal opacity after either bleogen pB1, or EGF treatment.

To provide further insights into the bleogen pB1-mediated healing effects, we performed pathway enrichment analysis on the transcriptome profiling of the EGF- and the bleogen pB1-treated keratinocytes. Our results suggest that both EGF and bleogen pB1 regulate the “TGF-β regulation of extracellular matrix”, the “interferon-alpha/beta signaling”, the “interferon signaling”, the “immune system signaling by interferons, interleukins, prolactin, and growth hormones”, and the “oncostatin M” pathways. Majority of the bleogen pB1’s DEGs involved in immune response were also affected by EGF, including STAT1 and STAT2 (Supplementary Figure S3). In addition, we identified 11 DEGs common to both bleogen pB1 and EGF within the “TGF-β regulation of extracellular matrix” pathway. Examples include CYR61, PTGS1, CDH5, and TNFSF10 (Supplementary Figure S3). Together, these results support that bleogen pB1 and EGF share, in part, similar pathways to regulate immune response signaling and the TGF-β-extracellular matrix, which could be the underlying reasons for their potential benefits in reducing corneal opacity during corneal healing (Qazi et al., 2010; Tandon et al., 2010; Wilson et al., 2012; Frangogiannis, 2020).

Consistent with our previous report, EGF and bleogen pB1 are functionally similar in terms of their capacity to promote an efficient corneal wound healing (Loo et al., 2021a). However, the main differences reside in their sequences and structures. The mammalian EGF and its related family members are characterized by a typical EGF domain with a two-subdomain structure. Unlike EGF and its related family, bleogen pB1 has a single domain and a cystine-knotted structure. Similar to other cysteine-rich peptides and due to its highly compact cystine-knot scaffold, bleogen pB1 is more than 100 times more resistant to proteolysis than the mammalian EGF (Loo et al., 2016; Loo et al., 2017; Tam et al., 2018; Kam et al., 2019b; Loo et al., 2021a; Loo et al., 2021b; Loo et al., 2022). Structural stability and proteolytic-resistant properties are highly desirable features for growth factors: they reduce their degradation and improve their cost-effectiveness, formulation feasibility, shelf life, and commercial viability for the development of ophthalmic formulations for corneal injury. Furthermore, the innate antifungal properties of bleogen pB1 are an additional advantage (Loo et al., 2017). Although this current study does not showcase the superior proteolytic stability and antifungal properties of bleogen pB1 compared to EGF in corneal wound healing, future studies using alternative animal models (e.g., involving the study of cornea infection and the employment of diabetic models) are warranted (Kernacki et al., 1997; Dong et al., 2005; Saghizadeh et al., 2005).

In conclusion, this study has expanded and demonstrated that bleogen pB1, like EGF, can accelerate the rat corneal wound healing and reduce prolonged inflammation and myofibroblast accumulation. The discovery of bleogen pB1 has generated new opportunities for the development of efficient corneal wound healing drugs and biomaterials that can reduce the likelihood of corneal scarring and permanent opacification. More importantly, the present study provides further evidence that bleogen pB1, as a bioactive miniprotein from *Pereskia bleo*, in part, is responsible for its traditional use in wounds and related ailments.

## Methods

### Materials

All chemicals and solvents, unless otherwise stated, were purchased from Sigma-Aldrich (United States) and Fisher Scientific (United States). Animal-Free Recombinant Human EGF was obtained from Peprotech (AF-100-15, United States).

### Solid-phase peptide synthesis and oxidative folding of bleogen pB1

Synthetic bleogen pB1 with N-terminal pyroglutamate was synthesized by Fmoc-based solid-phase peptide synthesis on HMPB-ChemMatrix resin (727741, Sigma-Aldrich, United States), by using a microwave-assisted peptide synthesizer as previously described in detail (Kam et al., 2019a; Loo et al., 2021a). The synthesized peptides were cleaved by using a cleavage cocktail (92.5% TFA, 2.5% H_2_O, 2.5% 1,2-ethanedithiol, 2.5% triisopropylsilane) for 1 h, at room temperature. The crude cleaved products were then folded under the following conditions: 10% dimethyl sulfoxide (DMSO), 90% 0.1 M NH_4_HCO_3_ at pH 8.0, and cystamine (10 equivalents) and cysteamine (100 equivalents) for 1 h, at room temperature. The folded pB1 was purified by preparative HPLC (250 × 21 mm; 5 μm; Phenomenex, United States) and identified by using MALDI-TOF mass spectrometry. Linear gradients of mobile phase A (0.1% TFA/H_2_O) and mobile phase B [0.1% TFA/acetonitrile (ACN)] were used. RP-HPLC, cell proliferation, and TR-FRET-based EGFR competitive displacement assays were performed in order to demonstrate the integrity and function of the synthetic bleogen pB1, as compared to its native form. The native bleogen pB1 was extracted from the leaves of *Pereskia bleo*, as has been previously described (Loo et al., 2017).

### Cell culture

HaCaT (human keratinocyte) cells were cultured in Dulbecco’s Modified Eagle medium (Gibco, United States) supplemented with 10% fetal bovine serum and 100 U/ml of penicillin and streptomycin (Gibco, United States).

### Cell proliferation assay

Cell proliferation was determined by using crystal violet staining as previously described (Loo et al., 2021a). Briefly, 1.0 × 10^4^ HaCaT cells were seeded into a 96-well plate with bleogen pB1 or EGF (positive control) in a serum-free medium. After the incubation period, the wells were fixed with 4% buffered paraformaldehyde for 20 min. The cells were then stained with 0.25% crystal violet in 20% methanol for 15 min. The excess stain was removed by rinsing with distilled water four to five times and by air-drying. Glacial acetic acid (10%) in Milli-Q water was then added in order to extract the crystal violet stain. The absorbance was then measured at 595 nm by using a microplate reader (Tecan Infinite^®^ 200 Pro, Switzerland).

### TR-FRET EGFR ligand binding assay

A competitive displacement assay was performed by using the EGF-EGFR LANCE Ultra TR-FRET Binding Kit, based on the manufacturer’s instructions (TRF1366C, PerkinElmer, United States). In this assay, streptavidin was conjugated with LANCE Europium chelate that binds to biotin-EGF, whereas EGFR-Fc interacts with anti-human IgG that was labeled with a ULight™ dye. Briefly, different concentrations of bleogen pB1 or EGF were mixed with the working solution and were incubated at room temperature for 2 h. The TR-FRET ratio was measured by using a microplate reader in dual emission mode (excitation: 340 nm, emission: 665 and 615 nm) (Cytation 1, Biotek, United States). The results are presented as the relative binding percentage of biotin-EGF, where EGF was used as positive control.

### Total RNA extraction, transcriptome sequencing, and RNA-seq data analysis

The total RNA was extracted from HaCaT keratinocytes by using the PureLink™ RNA Mini Kit (Thermo Fisher Scientific, MA, United States) according to the manufacturer’s instructions. Total RNA was assessed for quality and was quantified using a NanoDrop and an Agilent 2100 bioanalyzer (Supplementary Figure S3) (Thermo Fisher Scientific, MA, United States). Total RNA was submitted to Beijing Genomics Institute (BGI) for transcriptome sequencing by using the BGISEQ500 platform (BGI, China). The RNA-seq data were analyzed through an online system (Tom) provided by BGI. RNA-seq reads were then mapped to a human reference genome (GRCh38. p12). The DESeq2 software was used for the identification of DEGs. The parameters for identifying differentially expressed transcripts included a Q-value ≤ 0.05 and |log2FC|  ≥ 0.5. Pathway enrichment analysis was undertaken for the DEGs by using the BioPlanet 2019 database and the enrichR bioinformatics package.

### Acute corneal alkali wounding of rats

Male Wistar rats (8-week-old, 240.6 ± 8.0 g) (License No: SCXK (Zhe) 2019-0001; Certificate NO: 20211203Aazz0619000539) were purchased from Zhejiang Vital River Laboratory Animal Technology Co., Ltd. The rats were acclimatized for 1 week prior to the undertaking of the herein described experiments. Euthanasia was performed by using carbon dioxide inhalation, and all efforts were made in order to minimize animal suffering. All *in vivo* experimental procedures were approved by the institutional animal care and use committee and were performed at HD Biosciences, China. The AUF number for this study at HD Biosciences was 194.

The acute corneal alkali-induced wounding of rats was performed as previously described (Choi et al., 2017; Hakami et al., 2020). At the beginning of the study, randomization was performed based on body weight prior to the induction of the injury. An acute alkali burn was applied on the right eye of the rats by placing a 3.2-mm-diameter filter paper soaked in 1 N NaOH on the eye, for 30 s under general 0.3% isoflurane anesthesia. After a 30-s exposure, the corneas were rinsed with 40 ml of normal saline. All animals were randomly assigned into five corneal injury groups: 1) saline control (10 rats), 2) EGF (25 ng/ml; 10 rats), 3) pB1 (0.5 μg/ml; 10 rats), 4) pB1 (5 μg/ml; 10 rats), and 5) pB1 (12.5 μg/ml; 10 rats). Each compound was administered (at a volume of 10 µl) to the right eye of each corneal injury group from day 0 to day 4.

### Microscopic examination and fluorescein staining

Gross examination and photography of the corneas were performed on days 0, 3, 7, 10, and 14 using a handheld slit light under isoflurane anesthesia. On days 0 and 7, and after microscopic examination, 10 µl of 1% fluorescein sodium solution was applied onto the surface of the rats’ cornea and was left to incubate for approximately 3 min. After staining, the eyes were rinsed with normal saline in order to remove excess fluorescein and were then examined by using a cobalt blue light. The grading of fluorescein staining, the corneal opacity, and the corneal neovascularization were performed independently by experienced ophthalmologists that were blinded to the allocation of the animals in each group. The degree of fluorescein staining was scored by employing the following 0–4 numerical scale: 0, none; 1, area ≤ 1/4 quadrant; 2, 1/4 < area < 1/2 quadrant; 3, 1/2 < area < 3/4 quadrant; and 4, area > 3/4 quadrant. The degree of corneal opacity was scored by employing the following 0–4 numerical scale: 0, completely clear; 1, slightly hazy, iris and pupils easily visible; 2, slightly opaque, iris and pupils still detectable; 3, opaque, pupils hardly detectable; and 4, completely opaque with no view of the pupils. The degree of corneal neovascularization was scored by employing the following 0–4 numerical scale: 0, no neovascularization; 1, neovascularization in the peripheral cornea within one-third of the corneal diameter (as measured from the limbus); 2, neovascularization within two-thirds of the diameter (as measured from the limbus); 3, neovascularization within two-thirds of the diameter (as measured from the limbus); and 4, neovascularization observed over the entire cornea.

### Tissue collection for pathology

On day 14 after the induction of the acute alkali-induced corneal injury, all animals were euthanized with CO_2_ at a rate of 8 L/min. The right eyes were carefully collected and fixed in 10% neutral buffered formalin and paraffin embedded.

### H&E staining

Sections (4 µm thick) were stained with hematoxylin (#BA4041, BASO, China) and eosin (#BA4022, BASO, China) solution. H&E-stained slides were scanned with a Leica Aperio CS2 Scanner at ×200 magnification (Leica, United States).

### Immunohistochemical staining and analysis

Sections (4 µm thick) were placed on slides. After overnight drying, the paraffin was removed with xylene. The sections were then placed through a graded ethanol series and were immersed in distilled water. After heat-induced citrate antigen (pH 6.0) unmasking, the sections were immersed in a 3% hydrogen peroxide solution for 5 min. In order to avoid nonspecific staining, the sections were incubated in blocking serum (#X0909, DAKO, United States) for 15 min at room temperature, followed by an incubation with primary rabbit polyclonal anti-α-SMA (#ab5694, Abcam, United States; 1:600 dilution), primary rabbit polyclonal anti-Ki67 (#ab15580, Abcam, United States; 1:1500 dilution), primary mouse monoclonal anti-CD68 (#ab31630, Abcam, United States; 1:150 dilution), or primary mouse monoclonal anti-TNF-α (#ab220210, Abcam, United States; 1:800 dilution) antibodies for 1 h. The secondary goat polyclonal antibody that was conjugated to horseradish peroxidase (#K4003, DAKO, United States) was subsequently added and incubated. The sections were scanned with a Leica Aperio GT450 scanner (Leica, United States).

### Statistical analyses

Statistical comparisons were performed using GraphPad version 8.2.1 (United States). Data were analyzed through one-way analysis of variance, followed by Newman–Keuls *post hoc* tests. Data are expressed as the mean ± standard deviation, and a *p*-value < 0.05 was considered as statistically significant.

## Data Availability

The datasets presented in this study can be found in online repositories. The names of the repository/repositories and accession number(s) can be found below: NCBI Gene Expression Omnibus (GEO), GSE202512
