# Supplementary material for: Hyperstable EGF-like bleogen derived from cactus accelerates corneal healing in rats
Source: Front Pharmacol. 2022 Aug 16;13:942168. doi: 10.3389/fphar.2022.942168 (PMC9424907; doi:10.3389/fphar.2022.942168)
Supplement: Supplementary file 4 [file Table3.DOCX]

Supplementary Material

Ophthalmic application of a plant-derived peptidyl EGFR agonist accelerates corneal wound healing following acute alkali burn in rats

Shining Loo^1,#^, Antony Kam ^1,#^, James P. Tam^1,*^

^1^ School of Biological Sciences, Nanyang Technological University, Singapore 637551

^#^ These authors contribute equally to this work

*** Correspondence:**Professor James P. Tam, School of Biological Sciences, Nanyang Technological University, 60 Nanyang Drive, 637551, Singapore. Email: JPTam@ntu.edu.sg

**
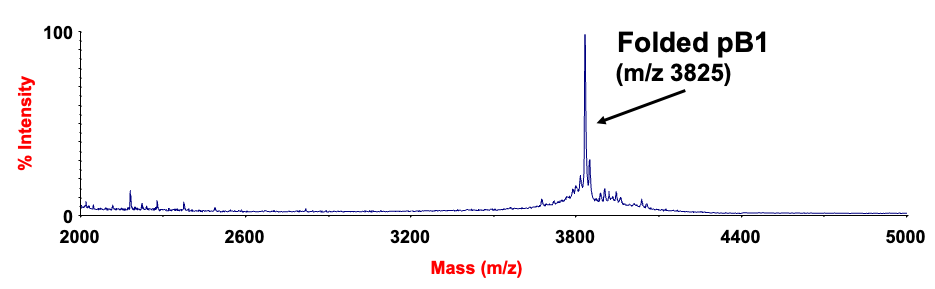
**

**Supplementary Figure S1.** MALDI-TOF MS profile of folded bleogen pB1.

**
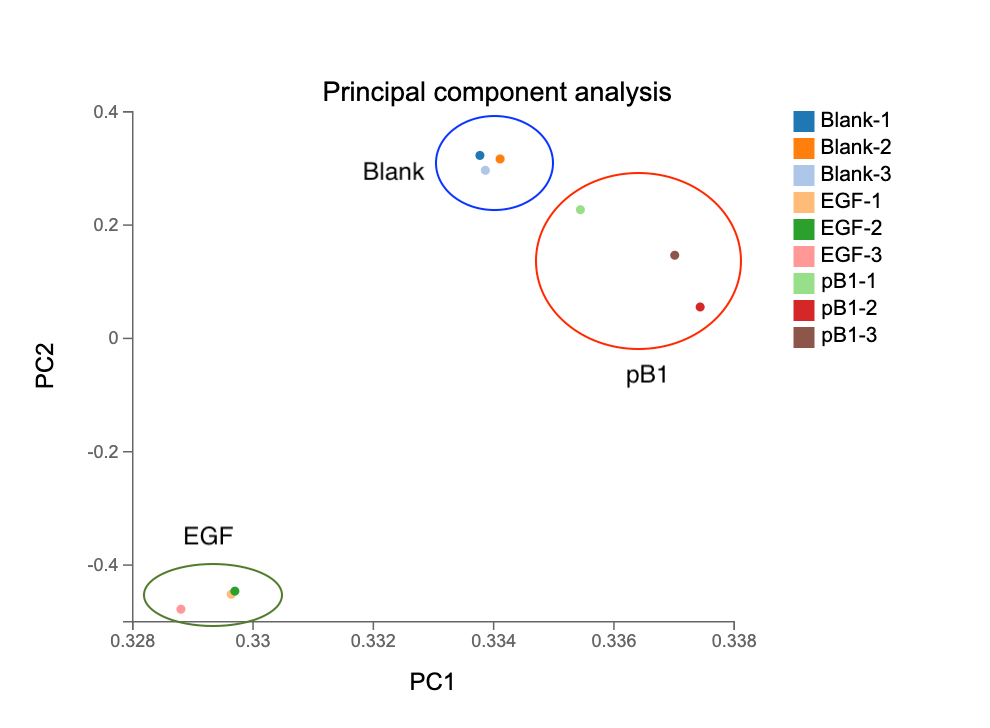
**

**Supplementary Figure S2.** Principal component analysis (PCA) of transcriptomics data.

| **Sample name** | **RIN/RQN** | **28S/18S** |
| --- | --- | --- |
| Blank-1 | 9.9 | 2 |
| Blank-2 | 9.9 | 1.9 |
| Blank-2 | 10 | 2.1 |
| EGF-1 | 10 | 2.1 |
| EGF-2 | 10 | 2.1 |
| EGF-3 | 10 | 2.1 |
| pB1-1 | 10 | 2 |
| pB1-2 | 10 | 2.2 |
| pB1-3 | 10 | 2 |

**Supplementary Figure S3.** RNA quality estimation data from Agilent 2100 bioanalyzer.
